# microRNA-140-3p protects hippocampal neuron against pyroptosis to attenuate sevoflurane inhalation-induced post-operative cognitive dysfunction in rats via activation of HTR2A/ERK/Nrf2 axis by targeting DNMT1

**DOI:** 10.1038/s41420-022-01068-4

**Published:** 2022-06-16

**Authors:** Zhiguo Wu, Jian Tan, Lichang Lin, Wenting Zhang, Wanqiu Yuan

**Affiliations:** 1 Department of Anesthesiology, Pingxiang People’s Hospital of Southern Medical University, Pingxiang, 337055 PR China; 2 Department of Vascular Interventional Surgery, Pingxiang People’s Hospital of Southern Medical University, Pingxiang, 337055 PR China

**Keywords:** Cell biology, Diseases

## Abstract

The incidence of post-operative cognitive dysfunction (POCD) remains a relatively prevalent complication in the elderly after surgery, especially in those receiving sevoflurane (Sevo) anesthesia. microRNA (miR)−140-3p has been demonstrated to orchestrate neuroinflammation and neuron apoptosis. However, the role of miR-140-3p in POCD remains largely unknown. In this context, this research was designed to explore whether miR-140-3p mediated Sevo inhalation-induced POCD in rats. A POCD rat model was established by Sevo inhalation, and a Sevo cell model was constructed in primary hippocampal neurons isolated from rats, followed by detection of miR-140-30 and HTR2A expression. Then, gain- and loss-of-function assays were implemented in rats and neurons. In rats, the cognitive function was evaluated by Water maze test and step-through test, and neuron apoptosis by TUNEL staining. In neurons, cell viability, apoptosis, and pyroptosis-related factors were tested by MTT, flow cytometry, and Western blot analysis respectively. Interaction between HTR2A and DNMT1 was assessed by MSP, and ChIP assay, and interaction between miR-140-3p and DNMT1 by dual-luciferase reporter assay, RIP and RNA pull-down. HTR2A and miR-140-3p were downregulated in POCD rats and Sevo-treated hippocampal neurons. Mechanistically, miR-140-3p negatively targeted DNMT1 to decrease HTR2A promoter methylation, thus upregulation HTR2A to activate ERK/Nrf2 pathway. miR-140-3p or HTR2A overexpression or activation of ERK/Nrf2 pathway elevated neuron viability and diminished their apoptosis and pyroptosis while alleviating Sevo-induced POCD in rats. Collectively, miR-140-3p might repress neuron pyroptosis to alleviate Sevo inhalation-induced POCD in rats via DNMT1/HTR2A/ERK/Nrf2 axis.

## Introduction

Sevoflurane (Sevo) is a volatile agent for maintaining clinical general anesthesia, which is well-tolerated for inhalation induction [1]. Nevertheless, Sevo inhalation can result in neuron apoptosis with pathological changes in brain hippocampus, triggering neurocognitive decline [2, 3]. Post-operative cognitive dysfunction (POCD) is a neurological complication that is linked to substantial morbidity and elevated mortality and especially frequently occurs in older people who have undergone surgical procedures under general anesthesia [4]. POCD is purported to include acute or persistent deficits in attention, concentration, learning, and memory after surgery [5]. Pyroptosis is an inflammatory form of programmed cell death that is caused by caspase-1/4/5/11 activated by some inflammasomes [6]. Cell pyroptosis assumes a critical role in the pathogenesis of POCD [7]. Therefore, it is of great significance for identifying a novel therapeutic target for restoration of cognitive function to elaborate the molecular mechanism behind cell pyroptosis in POCD.

The 5-hydroxytryptamine receptor 2A (HTR2A) can be epigenetically orchestrated through DNA methylation, which impacts infant brain development and adult cognitive function [8]. For instance, HTR2A methylation is correlated with infant neurobehavioral outcomes [9]. Besides, HTR2A can exert neuroprotective effects by activating the extracellular-signal-regulated kinase (ERK) pathway in neuroblastoma [10]. Moreover, ERK activation was previously documented to alleviate Sevo-induced cognitive dysfunction [11]. Also, nuclear factor-E2-related factor 2 (Nrf2) downregulation is able to aggravate cognitive dysfunction during aging [12]. Furthermore, DNA methylation of HTR2A is capable of reducing HTR2A expression to facilitate cognitive dysfunction during schizophrenia development [13]. Furthermore, DNA methyltransferase-1 (DNMT1) expression is linked to methylation of HTR2A [14]. As previously displayed, DNMT1 upregulation was associated with sepsis-induced cognitive dysfunction in mice [15]. Notably, microRNA (miR)-140-3p upregulation is capable of diminishing DNMT1 expression in WL-2 cells [16]. Additionally, miR-140-3p overexpression can repress the inflammation and apoptosis of oxygen-glucose deprivation and reperfusion (OGD/R)-treated neurons [17].

In this context, we hypothesized that miR-140-3p assumed a central role in Sevo inhalation-induced POCD by orchestrating DNMT1/HTR2A/ERK/Nrf2 axis. Therefore, we conducted this research to confirm this hypothesis, thus providing novel insight into the mechanism underlying Sevo inhalation-induced POCD.

## Results

### A rat model with POCD induced by Sevo inhalation was successfully established

**Fig. 1:**
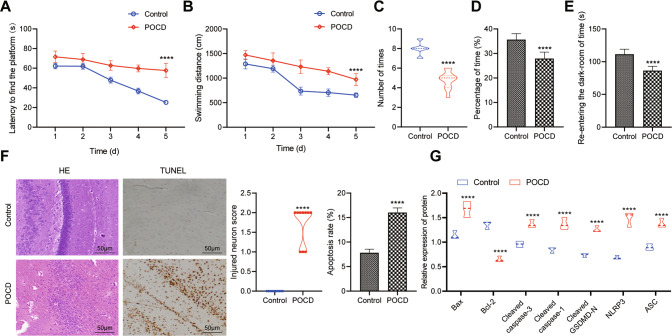
A POCD rat model is successfully induced. **A** Time for rats to find hidden platform in water maze after POCD modeling. **B** Swimming distance of rats before finding the platform hidden in the water maze after POCD modeling. **C** The times of rats crossing hidden platform in water maze after POCD modeling. **D** The residence time ratio of rats on the hidden platform of water maze after POCD modeling. **E** The time for rats to re-enter the darkroom in the step-through test after POCD modeling. **F** H&E staining (left) and TUNEL staining (right) for detecting pathological and apoptosis in the hippocampal tissues of rats after POCD modeling. **G** Western blot analysis to detect the protein expression of neuron apoptosis- and pyroptosis-related factors in hippocampal tissues after POCD modeling. There were ten rats in each group. *p < 0.05, **p < 0.01, and ***p < 0.001 vs. the rats without Sevo inhalation.

In order to study the influence of Sevo on POCD, 7-day-old rats were treated with or without Sevo inhalation. Then, water maze test was conducted when rats aged 6 weeks, which discovered significantly increased latency and swimming distance before rats found the hidden platform in the water maze (Fig. 1A, B) but distinctly decreased times of crossing the platform and time of rats spent in the target quadrant (Fig. 1C, D) after POCD modeling. Meanwhile, step-through test when rats were 3 months old reported the time for POCD rats to re-enter the darkroom was potently reduced (Fig. 1E).

Hematoxylin and eosin (H&E) and TUNEL staining showed that the levels of hippocampal neuron damage and apoptosis enhanced clearly in POCD rats (Fig. 1F). The Western blot results expounded that the expression of apoptosis- (Bax and Cleaved caspase-3) and pyroptosis-related proteins (Cleaved caspase-1, Cleaved-GSDMD, NLRP3, and ASC) in hippocampal tissues of POCD rats were sharply augmented, while Bcl-2 expression was remarkably reduced in rat hippocampal tissues (Fig. 1G). Taken together, the cognitive function of rats was damaged by Sevo inhalation, and POCD rat model was established successfully.

### HTR2A overexpression elevated the viability and inhibited the apoptosis and pyroptosis of hippocampal neurons from POCD rats

**Fig. 2:**
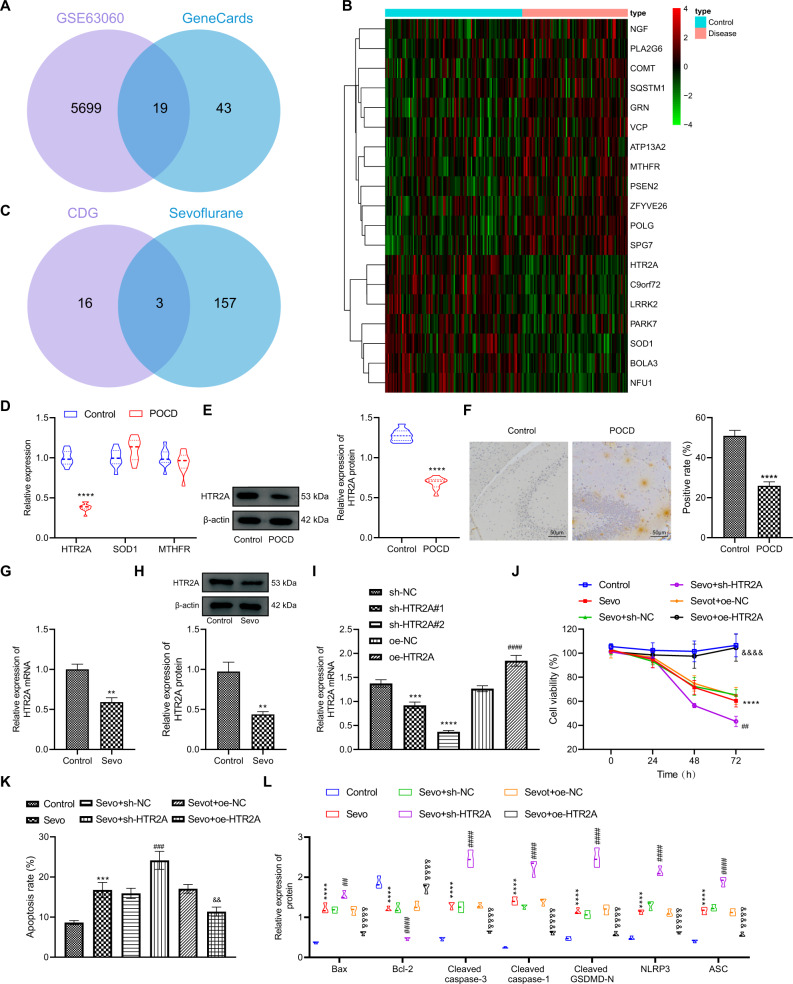
HTR2A upregulation induces the viability but suppresses the apoptosis and pyroptosis of hippocampal neurons from POCD rats. **A** Venn map of intersection of the differentially expressed genes in GSE63060 with CDGs retrieved from GeneCards database. **B** Heat maps of 19 differentially expressed CDGs between 104 control samples and 80 POCD samples in GSE63060 dataset. **C** Venn map of intersection of 19 differentially expressed CDGs with the targets of Sevo retrieved from GeneCards database. **D** The detection of expression of HTR2A, SOD1, and MTHFR in the hippocampal tissues of rats after POCD modeling by RT-qPCR. **E** The detection of protein expression of HTR2A in the hippocampal tissues of rats after modeling by Western blot analysis. **F** The detection of expression of HTR2A in the hippocampal tissues of rats after modeling by immunohistochemistry. **G** RT-qPCR to detect the expression of HTR2A in the hippocampal neurons after Sevo treatment. **H** Western blot analysis for detecting the protein expression of HTR2A in the hippocampal neurons after Sevo treatment. **I** RT-qPCR to detect the gene expression of HTR2A in hippocampal neurons after sh-HTR2A or oe-HTR2A treatment. **J** MTT to detect the viability of hippocampal neurons after treatment with Sevo and/or sh-HTR2A or oe-HTR2A. **K** The apoptosis of hippocampal neurons detected by flow cytometry after treatment with Sevo and/or sh-HTR2A or oe-HTR2A. **L** Western blot analysis to measure the protein expression of apoptosis- and pyroptosis-related factors after treatment with Sevo and/or sh-HTR2A or oe-HTR2A. In **D**–**H**, *p < 0.05, **p < 0.01, ***p < 0.001 vs. the rats with POCD. In **I**, *p < 0.05, **p < 0.01, and ***p < 0.001 vs. the neurons treated with Sevo + sh-NC; ^#^*p* < 0.05, ^##^*p* < 0.01, and ^###^*p* < 0.001 vs. the neurons treated with Sevo + oe-NC. In **J**–**L**, *p < 0.05, **p < 0.01, and ***p < 0.001 vs. the neurons treated without Sevo; ^#^*p* < 0.05, ^##^*p* < 0.01, and ^###^*p* < 0.001 vs. the neurons treated with Sevo + oe-NC; ^&^*p* < 0.05, ^&&^*p* < 0.01, and ^&&&^*p* < 0.001 vs. the neurons treated with Sevo + oe-NC. The experiment was repeated three times.

Next, the mechanism of POCD in rats was further studied. By analyzing the gene expression dataset GSE63060 of POCD patients and searching POCD-related genes (score *>*30) from GeneCards database, 19 cognitive dysfunction-related genes (CDGs) were found (Fig. 2A, B). Then, these CDGs were intersected with targets of Sevo predicted by GeneCards database, which obtained three intersected target genes encompassing SOD1, MTHFR, and HTR2A (Fig. 2C). RT-qPCR exhibited that in POCD rats, only HTR2A was dramatically downregulated, while SOD1 and MTHFR levels were not substantially changed (Fig. 2D). Therefore, we chose HTR2A as the follow-up research object.

Subsequently, Western blot analysis and immunohistochemistry results demonstrated that HTR2A was significantly downregulated in hippocampal tissues of POCD rats (Fig. 2E, F). Next, hippocampal neurons were treated with Sevo, and RT-qPCR and Western blot analysis offered data displaying that HTR2A expression in hippocampal neurons was memorably reduced after Sevo treatment (Fig. 2G, H). Therefore, we chose HTR2A as the subject for subsequent study.

Then, we explored how HTR2A affected the viability, apoptosis, and pyroptosis of rat hippocampal neurons. First, the efficiency of sh-HTR2A and oe-HTR2A in hippocampal neurons was detected by RT-qPCR, which illustrated that HTR2A expression in neurons treated with sh-HTR2A#1 or sh-HTR2A#2 was strikingly lowered, with the lowest expression in neurons treated with sh-HTR2A#2. Therefore, the follow-up experiments were conducted with sh-HTR2A#2 (sh-HTR2A). In addition, HTR2A expression in neurons after oe-HTR2A treatment was noticeably enhanced (Fig. 2I). MTT assay and flow cytometry depicted that hippocampal neuron viability was strikingly restrained and their apoptosis was memorably facilitated after treatment with Sevo, which was promoted by further sh-HTR2A treatment but was negated by further oe-HTR2A treatment (Fig. 2J, K). Meanwhile, treatment with Sevo contributed to noticeably increased expression of pro-apoptotic proteins Bax and Cleaved caspase-3 and pyroptosis-related proteins, namely Cleaved caspase-1, Cleaved GSDMD, NLRP3, and ASC but decreased expression of anti-apoptotic protein Bcl-2 in rat hippocampal neurons; this effect was promoted by additional sh-HTR2A treatment but was negated by additional oe-HTR2A treatment (Fig. 2L). Collectively, silencing HTR2A could accelerate the viability and repress the apoptosis and pyroptosis of rat hippocampal neurons from POCD rats.

### ERK/Nrf2 pathway was inhibited in POCD rats while HTR2A activated it in hippocampal neurons of POCD rats

**Fig. 3:**
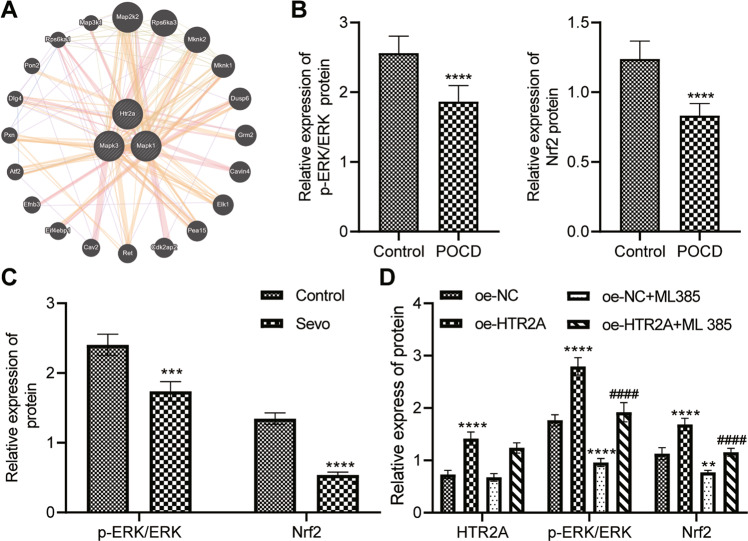
HTR2A overexpression results in activation of ERK/Nrf2 pathway in hippocampal neurons of POCD rats. **A** Bioinformatics software predicted that HTR2A was involved in ERK/Nrf2 pathway. **B** Western blot analysis of the protein expression of Nrf2 and the phosphorylated ERK/total ERK protein expression ratio in the hippocampal tissues (10 rats/group, *p < 0.05, **p < 0.01, and ***p < 0.001 vs. the rats without Sevo inhalation). **C** Western blot for determining the protein expression of and Nrf2 and phosphorylated ERK/total ERK protein expression ratio in rat hippocampal neurons (The experiment was repeated three times; *p < 0.05, **p < 0.01, and ***p < 0.001 vs. the rats without Sevo inhalation). **D** The *p*rotein expression of HTR2A and Nrf2 and phosphorylated ERK/total ERK protein expression ratio in rat hippocampal neurons by Western blot analysis (The experiment was repeated three times; *p < 0.05, **p < 0.01, and ***p < 0.001 vs. the neurons treated with oe-NC; ^#^*p* < 0.05, ^##^*p* < 0.01, and ^###^*p* < 0.001 vs. the neurons with oe-HTR2A treatment).

Then, we ascertained the downstream mechanism of HTR2A in POCD. Furthermore, bioinformatics software predicted that HTR2A was involved in the orchestration of ERK/Nrf2 pathway (Fig. 3A). Therefore, we further investigated whether HTR2A could manipulate the ERK/Nrf2 pathway in POCD. Western blot analysis results described significantly decreased Nrf2 protein expression and ERK phosphorylation level in the hippocampal tissues of POCD rats as well as in the hippocampal neurons with Sevo treatment (Fig. 3B, C).

Afterwards, HTR2A was overexpressed in hippocampal neurons and ERK/Nrf2 pathway inhibitor ML385 was added. The protein expression of HTR2A and Nrf2 and the phosphorylation level of ERK in hippocampal neurons were clearly increased after oe-HTR2A treatment, while in the presence or absence of oe-HTR2A, ML385 treatment did not affect HTR2A protein expression in hippocampal neurons, but sharply reduced the Nrf2 protein expression and the phosphorylation level of ERK (Fig. 3D). In conclusion, ERK/Nrf2 pathway was blocked in POCD rats whilst HTR2A activated it in POCD rats.

### DNMT1 was highly expressed in POCD rats and promoted the methylation of HTR2A to dampen its transcription in hippocampal neurons of POCD rats

**Fig. 4:**
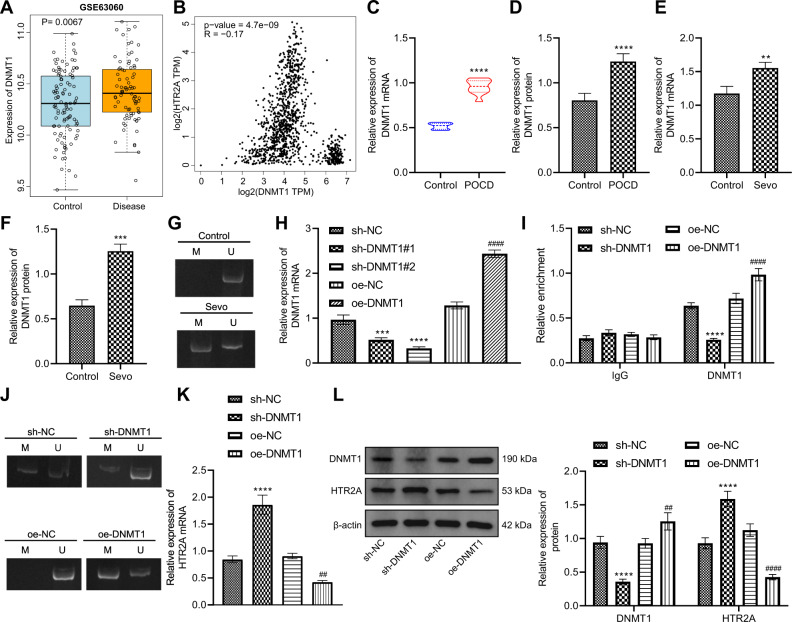
DNA methyltransferase DNMT1 induces HTR2A methylation to repress its transcription in hippocampal neurons of POCD rats. **A** Expression of DNMT1 in POCD samples of GSE63060 (104 control samples and 80 POCD samples, *p < 0.05, **p < 0.01, and ***p < 0.001 vs. control samples). **B** GEPIA database to analyze the expression of DNMT1 and HTR2A in brain tissue of GTEx. **C** Detection of expression of DNMT1 gene in the hippocampal tissues of rats after POCD modeling by RT-qPCR (*p < 0.05, **p < 0.01, and ***p < 0.001 vs. the rats without POCD). **D** Western blot analysis of the protein expression of DNMT1 in the hippocampal tissues of rats after POCD modeling (*p < 0.05, **p < 0.01, and ***p < 0.001 vs. the rats without POCD). **E** Detection of the ex*p*ression of DNMT1 gene in rat hippocampal neurons after Sevo treatment by RT-qPCR (*p < 0.05, **p < 0.01, and ***p < 0.001 vs. the neurons without Sevo treatment). **F** Western blot analysis to detect the protein expression of DNMT1 in rat hippocampal neurons after Sevo treatment (*p < 0.05, **p < 0.01, and ***p < 0.001 vs. the neurons without Sevo treatment). **G** MSP for measuring the methylation level of HTR2A promoter in hippocampal neurons after Sevo treatment. **H** RT-qPCR to determine the expression of DNMT1 gene in hippocampal neurons after sh-DNMT1 or oe-DNMT1 treatment (*p < 0.05, **p < 0.01, and ***p < 0.001 vs. the neurons treated with sh-NC; ^#^*p* < 0.05, ^##^*p* < 0.01, and ^###^*p* < 0.001 vs. the neurons treated with oe-NC). **I** Detection of enrichment of DNMT1 in HTR2A promoter by ChIP assay after sh-DNMT1 or oe-DNMT1 treatment. **J** MSP to detect the methylation level of HTR2A promoter in hippocam*p*al neurons after sh-DNMT1 or oe-DNMT1 treatment. **K** RT-qPCR to detect the gene expression of HTR2A in hippocampal neurons after sh-DNMT1 or oe-DNMT1 treatment. **L** Western blot analysis for detecting the protein expression of DNMT1 and HTR2A in hippocampal neurons after sh-DNMT1 or oe-DNMT1 treatment. In **I**, **K**, and **L**, *p < 0.05, **p < 0.01, and ***p < 0.001 vs. the neurons treated with sh-NC; ^#^*p* < 0.05, ^##^*p* < 0.01, and ^###^*p* < 0.001 vs. the neurons treated with oe-NC. The cell experiment was repeated three times.

Next, the focus of our research was shifted to the upstream mechanism of HTR2A in POCD. From GSE63060 data, we found that DNMT1 was highly expressed in POCD patients (Fig. 4A). Gene Expression Profiling Interactive Analysis (GEPIA) database manifested that DNMT1 and HTR2A expression shared a negative correlation in brain tissue data of Genotype-Tissue Expression (GTEx) (Fig. 4B). Therefore, we speculated that DNMT1 might mediate HTR2A expression by promoting HTR2A promoter methylation.

As reflected by RT-qPCR and Western blot data, DNMT1 expression in hippocampal tissues of POCD rats increased remarkably (Fig. 4C, D). A consistent increase was detected in primary rat hippocampal neurons treated with Sevo (Fig. 4E, F). Subsequently, MSP demonstrated that after Sevo treatment, the methylation level of CpG island in HTR2A promoter region in hippocampal neurons was markedly elevated (Fig. 4G).

Next, the efficiency of sh-DNMT1 and oe-DNMT1 in primary rat hippocampal neurons was detected by RT-qPCR, which demonstrated that DNMT1 expression was significantly lowered by sh-DNMT1#1 or sh-DNMT1#2, especially sh-DNMT1#2; therefore, sh-DNMT1#2 (sh-DNMT1) was selected for subsequent experimentation. In addition, DNMT1 expression was obviously enhanced by oe-DNMT1 (Fig. 4H).

Furthermore, ChIP assay showed that the enrichment level of DNMT1 in HTR2A promoter region was potently diminished by treatment with sh-DNMT1, which was contrary after treatment with oe-DNMT1 (Fig. 4I). Then MSP result documented that the methylation level of HTR2A promoter decreased observably after sh-DNMT1 treatment, while the opposite result was observed after treatment with oe-DNMT1 (Fig. 4J). As indicated by RT-qPCR and Western blot analysis results, HTR2A expression was enhanced distinctly after sh-DNMT1 treatment, which was opposite after oe-DNMT1 treatment (Fig. 4K, L).

Conclusively, DNMT1 was highly expressed in POCD rats, and it could promote the methylation of HTR2A and inhibit its transcription in hippocampal neurons of POCD rats.

### miR-140-3p, a downregulated miRNA in POCD rats, negatively targeted DNMT1 in hippocampal neurons

**Fig. 5:**
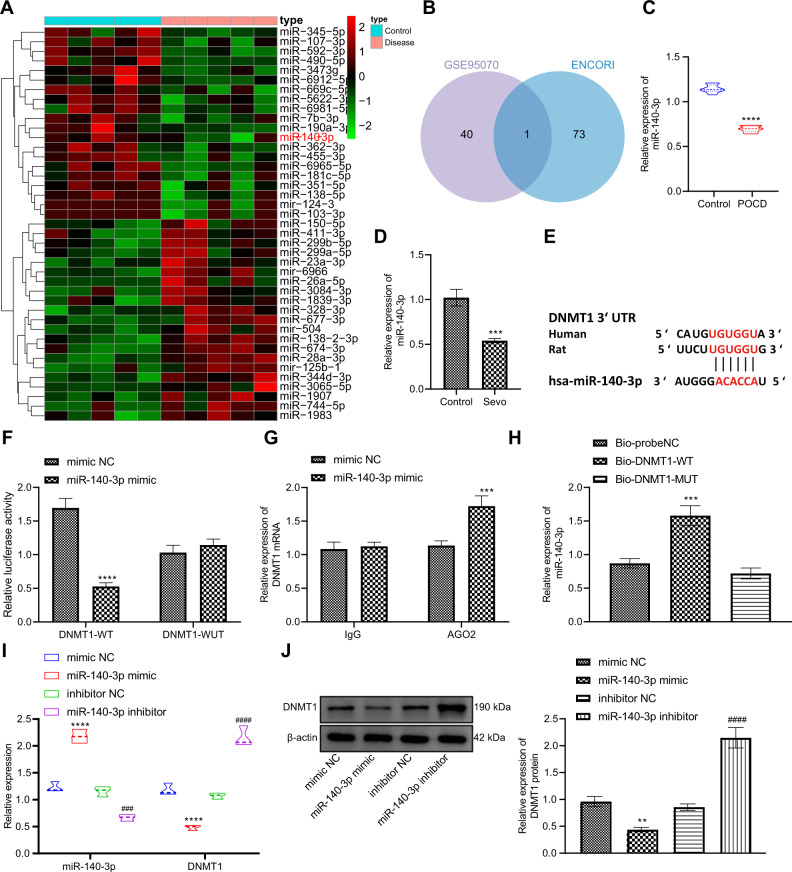
DNMT1 is negatively targeted by miR-140-3p in rat hippocampal neurons. **A** The heat map showing low expression of miR-140-3p in GSE95070 in POCD samples. **B** Venn map of intersection of the predicted miRNAs targeting DNMT1 in ENCORI8 database with the differentially expressed miRNAs in GSE95070. **C** The detection of expression of miR-140-3p in the hippocampal tissues of rats after POCD modeling by RT-qPCR (There were ten rats in each group. *p < 0.05, **p < 0.01, and ***p < 0.001 vs. the rats without POCD). **D** The detection of expression of miR-140-3p in hippocampal neurons after Sevo treatment by RT-qPCR (The experiment was repeated three times; *p < 0.05, **p < 0.01, and ***p < 0.001 vs. the control neurons). **E** The prediction of binding sites of miR-140-3p and DNMT1 by ENCORI database. **F** Dual-luciferase reporter assay for assessing the targeted binding of miR-140-3p and DNMT1 (The experiment was repeated three times; *p < 0.05, **p < 0.01, and ***p < 0.001 vs. mimic NC treatment). **G** The detection of interaction between miR-140-3p and DNMT1 by RIP assay (The experiment was repeated three times; *p < 0.05, **p < 0.01, and ***p < 0.001 vs. mimic NC treatment). **H** The analysis of interaction between miR-140-3p and DNMT1 by RNA pull-down (The experiment was repeated three times; *p < 0.05, **p < 0.01, and ***p < 0.001 vs. Bio-DNMT1-MUT and Bio-probeNC). **I** The detection of expression of miR-140-3p and DNMT1 in hippocampal neurons after miR-140-3p mimic or inhibitor treatment by RT-qPCR. **J** Western blot analysis to detect the protein expression of DNMT1 in hippocampal neurons after miR-140-3p mimic or inhibitor treatment. In **I**, **J**, the experiment was repeated three times; *p < 0.05, **p < 0.01, and ***p < 0.001 vs. the neurons treated with mimic NC, ^#^*p* < 0.05, ^##^*p* < 0.05, and ^###^*p* < 0.05 vs. the neurons treated with inhibitor NC.

In order to further explore whether DNMT1 may be targeted by miRNAs, the miRNAs targeting DNMT1 were predicted from Encyclopedia of RNA Interactomes (ENCORI) database and were intersected with POCD-related miRNA expression dataset (GSE95070), revealing miR-140-3p in the intersection, and the heat map showed the downregulation of miR-140-3p in POCD samples (Fig. 5A, B). As indicated by RT-qPCR, miR-140-3p expression was potently reduced in both the hippocampal tissues of POCD rats and the Sevo-induced hippocampal neurons (Fig. 5C, D).

We further investigated whether miR-140-3p could directly orchestrate DNMT1 expression. Firstly, the binding sites between miR-140-3p and DNMT1 were obtained from ENCORI database (Fig. 5E). Subsequently, dual-luciferase reporter assay displayed that the luciferase activity of cells with DNMT1-WT was distinctly decreased by miR-140-3p mimic, while that of cells with DNMT1-MUT remained almost unchanged (Fig. 5F), which suggested that miR-140-3p could target DNMT1. RIP results exhibited that DNMT1 enrichment in the samples pulled down by AGO2 was noticeably augmented after miR-140-3p gain-of-function (Fig. 5G), which indicated that miR-140-3p might interact with DNMT1. RNA pull-down assay also described that miR-140-3p expression was clearly enhanced by Bio-DNMT1-WT (Fig. 5H).

Next, we further studied the regulation of DNMT1 by miR-140-3p. RT-qPCR and Western blot results illustrated that miR-140-3p expression was markedly increased following treatment with miR-140-3p mimic, while DNMT1 expression was notably reduced, which was opposite upon miR-140-3p inhibition (Fig. 5I, J).

In brief, miR-140-3p was underexpressed in POCD rats, and it could target DNMT1 in hippocampal neurons of POCD rats.

### miR-140-3p accelerated hippocampal neuron viability and restrained their apoptosis and pyroptosis via DNMT1/HTR2A/ERK/Nrf2 axis

**Fig. 6:**
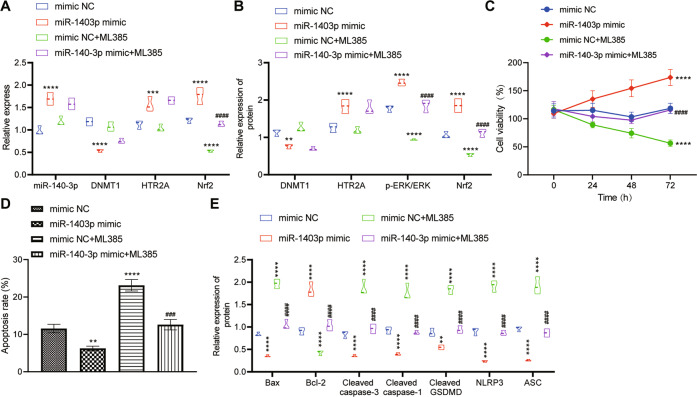
miR-140-3p manipulates the DNMT1/HTR2A/ERK/Nrf2 axis to facilitate the viability and repress the apoptosis and pyroptosis of rat hippocampal neurons. Hippocampal neurons were transduced with mimic NC, miR-140-3p, mimic NC + ML385, or miR-140-3p + ML385. **A** The detection of expression of miR-140-3p, DNMT1, HTR2A, and Nrf2 in hippocampal neurons by RT-qPCR. **B** The detection of protein expression of DNMT1, HTR2A, and Nrf2 and phosphorylated ERK/total ERK protein expression ratio in hippocampal neurons by Western blot analysis. **C** MTT to detect the cell viability of hippocampal neurons. **D** The apoptosis of hippocampal neurons determined by flow cytometry. **E** Western blot analysis to detect the protein expression of apoptotic and pyrolytic factors in hippocampal neurons. The experiment was repeated three times. *p < 0.05, **p < 0.01, and ***p < 0.001 vs. the neurons treated with mimic NC; ^#^*p* < 0.05, ^##^*p* < 0.01, and ^###^*p* < 0.001 vs. the neurons treated with miR-140-3p mimic.

Next, we further explored whether miR-140-3p could modulate the viability, apoptosis and pyroptosis of hippocampal neurons through DNMT1/HTR2A/ERK/Nrf2 axis. Through RT-qPCR and Western blot analyses, the miR-140-3p, HTR2A, and Nrf2 expression and the phosphorylation level of ERK in Sevo-treated rat hippocampal neurons were detected to be markedly increased while that of DNMT1 was evidently decreased after miR-140-3p mimic treatment. However, in the presence of either mimic-NC or miR-140-3p mimic, no significant difference regarding miR-140-3p, DNMT1, and HTR2A expression in the Sevo-induced rat hippocampal neurons was noted after treatment with ML385, but the phosphorylation level of ERK and Nrf2 expression decreased substantially (Fig. 6A, B).

MTT and flow cytometry documented that ML385 treatment resulted in decline of cell viability and elevation of cell apoptosis of Sevo-induced hippocampal neurons. After miR-140-3p mimic treatment, the viability of Sevo-induced hippocampal neurons was dramatically enhanced and their apoptosis was significantly diminished, which was annulled by further ML385 treatment (Fig. 6C, D). In addition, Western blot analysis results of apoptosis- and pyroptosis-related factors in Sevo-induced hippocampal neurons were concordant with those of flow cytometry (Fig. 6E).

In summary, miR-140-3p might regulate the DNMT1/HTR2A/ERK/Nrf2 axis to increase the viability and reduce the apoptosis and pyroptosis of hippocampal neurons.

### miR-140-3p alleviated Sevo-induced POCD in rats by mediating DNMT1/HTR2A/ERK/Nrf2 axis

**Fig. 7:**
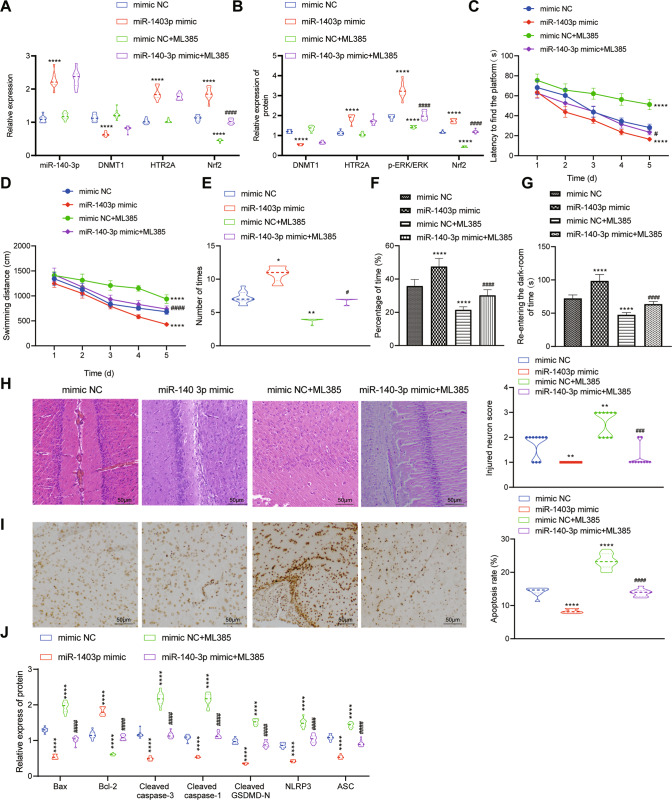
miR-140-3p modulates the DNMT1/HTR2A/ERK/Nrf2 axis to attenuate Sevo-induced POCD in rats. POCD rats were treated with mimic NC, miR-140-3p, mimic NC + ML385, or miR-140-3p + ML385. **A** The detection of expression of miR-140-3p, DNMT1, HTR2A, and Nrf2 in the hippocampal tissues of rats by RT-qPCR. **B** The detection of protein levels of DNMT1, HTR2A, and Nrf2 and phosphorylated ERK/total ERK protein expression ratio through Western blot analysis. **C** Time for rats to find hidden platform in water maze. **D** Swimming distance of rats before finding the hidden platform in water maze. **E** The times of rats crossing hidden platform in water maze. **F** The residence time ratio of rats on the hidden platform of water maze. **G** The time for rats to re-enter the darkroom in the step-through test. **H** H&E staining to determine the pathological changes of hippocampal tissues. **I** TUNEL staining to detect the apoptosis of hippocampal neurons in rats. **J** Western blot analysis to detect the protein expression of apoptosis- and pyroptosis-related factors in hippocampal tissues of rats. There were ten rats in each group. *p < 0.05, **p < 0.01, and ***p < 0.001 vs. the rats treated with mimic NC; ^#^*p* < 0.05, ^##^*p* < 0.01, and ^###^*p* < 0.001 vs. the rats treated with miR-140-3p mimic.

Finally, we further explored whether miR-140-3p could affect POCD induced by Sevo via the DNMT1/HTR2A/ERK/Nrf2 axis in vivo. RT-qPCR and Western blot analysis indicated that after miR-140-3p mimic treatment, miR-140-3p, HTR2A and Nrf2 expression as well as the phosphorylation level of ERK potently increased in the hippocampal tissues of POCD rats while DNMT1 expression decreased strikingly. No significant difference in miR-140-3p, DNMT1 and HTR2A expression but obvious declines of Nrf2 expression and ERK phosphorylation level were detected in hippocampal tissues of POCD rats after ML385 treatment in the presence of either mimic NC or miR-140-3p mimic (Fig. 7A, B).

Next, the results of water maze experiment showed that before finding the hidden platform in the water maze, the latency and swimming distance of POCD rats treated with miR-140-3p mimic were sharply reduced, and the times of crossing the platform and the time spent in the target quadrant were memorably increased while ML385 treatment triggered opposite trends; the effect of miR-140-3p mimic was nullified by ML385 treatment (Fig. 7C–F). Subsequently, the time of POCD rats treated with miR-140-3p mimic before re-entering the darkroom was obviously augmented, while that of POCD rats before re-entering the dark room was clearly diminished by ML385 in the presence of either mimic NC or miR-140-3p mimic (Fig. 7G).

Further H&E, TUNEL staining and Western blot analysis results presented that hippocampal neuron damage, apoptosis and pyroptosis of POCD rats were dramatically declined by miR-140-3p mimic but was evidently elevated after ML385 treatment; whereas, the impact induced by miR-140-3p mimic was abrogated by ML385 treatment (Fig. 7H–J).

Taken together, miR-140-3p might alleviate Sevo-caused POCD in rats via the DNMT1/HTR2A/ERK/Nrf2 axis.

## Discussion

POCD negatively influences subjective cognitive function and life quality in affected patients and also facilitates the risk of dementia and mortality [18]. Moreover, the incidence of POCD is increasing steadily in older patients after surgery, particularly in patients receiving Sevo anesthesia [19]. miR-140-3p has been displayed to be altered in breast cancer, hepatocellular carcinoma, lung carcinoma, and spinal chordoma [20]. However, the functionality of miR-140-3p in POCD remained enigmatic. Based on this, our research aimed at figuring out whether miR-140-3p orchestrated Sevo inhalation-induced POCD and the potential mechanism. Our data revealed that miR-140-3p might inversely target DNMT1 to inhibit the methylation of HTR2A promoter and promote the transcription of HTR2A, thus activating the ERK/Nrf2 pathway and then alleviating POCD induced by Sevo in rats.

Firstly, we observed that HTR2A was poorly expressed in hippocampal tissues of POCD rats. It has been recognized that HTR2A plays a crucial role in orchestration of neurological disorders, so its agonists hold therapeutic potential in neurological disorders [21]. For example, the research of Hasselbalch et al. uncovered that HTR2A downregulation participated in mild cognitive dysfunction in Alzheimer’s disease [22]. Besides, another research manifested that activation of HTR2A could attenuate cognitive dysfunction in the hemiparkinsonian rats [23]. Therefore, these findings suggested that HTR2A overexpression might be associated with alleviation of Sevo-induced POCD. Further data in our work exhibited that HTR2A increased viability and decreased apoptosis and pyroptosis of hippocampal neurons from POCD rats. Corroborating findings were reported in a prior work that HTR2A agonist (TCB-2) diminished streptozotocin-induced neuron apoptosis in the hippocampus of rats [24]. However, there has hitherto been little research about the role of HTR2A in cell pyroptosis. In addition, we also found that HTR2A upregulation restrained expression of pyroptosis-related genes (Cleaved-GSDMD, NLRP3, and ASC) in hippocampal neurons from POCD rats. As a critical component of the NLRP3 inflammasome, GSDMD is involved in NLRP3 inflammasome activation and undergoes proteolytic cleavage by caspase-1 to secrete its N-terminal fragment, which in turn modulates pyroptosis [25]. Cleaved-GSDMD forms membrane pores resulting in cytokine release and/or programmed lytic cell death, termed pyroptosis [26]. Additionally, ASC functions as an accelerator of cell pyroptosis [27]. Cumulatively, HTR2A upregulation contributed to suppression of neuron apoptosis and pyroptosis to alleviate Sevo-induced POCD in rats.

Moreover, it was previously reported that HTR2A activated ERK pathway to augment basal progenitor proliferation [28]. Consistently, we unveiled that HTR2A overexpression elevated viability and diminished apoptosis and pyroptosis of hippocampal neurons from POCD rats by activating the ERK/Nrf2 pathway. ERK pathway activation acts to alleviate anesthesia-induced cognitive dysfunction by enhancing neuron survival and neuron apoptosis [29]. Also, activating the Nrf2 pathway depresses cognitive dysfunction caused by Aβ_1-42_ in mice [30]. Meanwhile, cognitive dysfunction induced by chronic cerebral hypoperfusion in rats can be suppressed by activating the ERK/Nrf2 pathway [31]. Concordant with our results, it was noted in prior research that upregulation of ERK/Nrf2 pathway triggered suppression of neuron apoptosis in the hippocampus to ameliorate cognitive dysfunction induced by chronic cerebral hypoperfusion in rats [32]. Intriguingly, Nrf2 pathway activation can lead to inhibition of cell pyroptosis in a mouse model of renal ischemia-reperfusion injury, accompanied by reduced ASC, caspase-1, and GSDMD expression [33]. Notably, the work of Li et al. manifested that upregulation of ERK/Nrf2 pathway was able to curtail pyroptosis and apoptosis of neurons exposed to Sevo [34], which was concurrent with our findings.

There are extensive data demonstrating that HTR2A methylation assumes a pivotal role in fetal brain development and adult cognitive function [8]. In our research, methylation of the HTR2A promoter was observed in Sevo-triggered POCD in rats, and miR-140-3p negatively targeted DNMT1 to lower HTR2A methylation, thus upregulating HTR2A to augment viability and decrease apoptosis and pyroptosis of hippocampal neurons from POCD rats. Similarly, it has been elucidated that DNMT1 overexpression causes methylation of HTR2A promoter in myeloma cells [14] and that miR-140-3p overexpression is linked to decline of DNMT1 expression in WL-2 cells [16]. Furthermore, a prior work discovered that DNMT1 upregulation was involved in cognitive dysfunction induced by zinc deficiency in rats [35]. Besides, miR-140-3p overexpression protects neurons against apoptosis after OGD/R induction [17]. Also, it has been displayed that miR-140 promotes the cerebral protective effects of dexmedetomidine against hypoxic-ischemic brain damage in rats by repressing neuron apoptosis [36]. However, there are limited researches about the impact of miR-140-3p on POCD and cell pyroptosis. This background calls for further research to ascertain the specific role of miR-140-3p in cell pyroptosis.

**Fig. 8:**
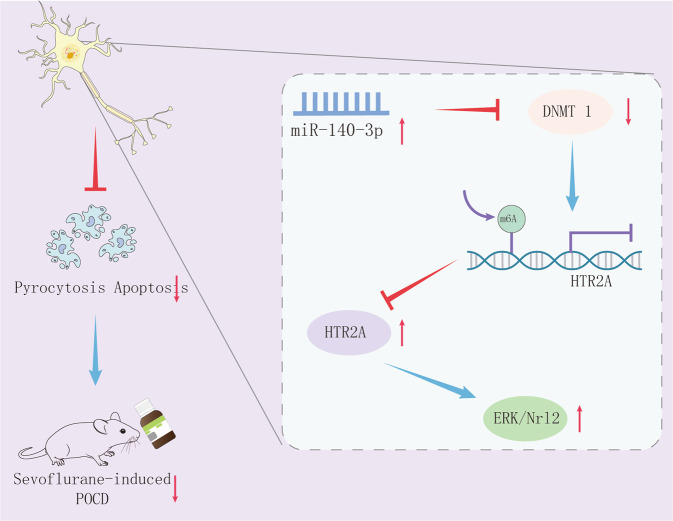
Mechanism of miR-140-3p diminishing hippocampal neuron pyroptosis to alleviate Sevo inhalation-induced POCD in rats via DNMT1/HTR2A/ERK/Nrf2 axis.

In conclusion, our data lent crucial support to the notion that miR-140-3p might facilitate neuron viability and restrain their apoptosis and pyroptosis to alleviate Sevo inhalation-induced POCD in rats via DNMT1/HTR2A/ERK/Nrf2 axis (Fig. 8). The discovery of miR-140-3p being a new manipulator that controls Sevo inhalation-induced POCD offers a fresh molecular insight into the following new therapy development for POCD. However, we failed to conduct a time-dependent experiment to screen the best therapeutic window of mR-140-30, which will be explored in the future study.

## Materials and methods

### Bioinformatics analysis

Gene expression dataset GSE63060 and miRNA expression dataset GSE95070 were acuired from Gene Expression Omnibus database. GSE63060 included 104 control samples and 80 POCD samples. GSE95070 included five control samples and five POCD samples. The R language “limma” package was used to identify differentially expressed genes in GSE63060 dataset with *p* value <0.01 as the threshold. In addition, the threshold of |log fold change (FC)|>1 and *p* value <0.05 was employed to seek the differentially expressed miRNAs in GSE95070 dataset. POCD- and Sevo-related targets were searched through Genecards database. GEPIA database was used to analyze the gene expression correlation in brain tissue data of GTEx.

### Establishment of rat model with POCD

As rodents were anesthetized on the 7th day after birth, they are more prone to anesthesia-induced neurodegeneration. Therefore, male Sprague-Dawley rats (Vitalriver, Beijing, China; weighing 14–18 g; aged 7 days) were available from the animal center of Shanghai Jiao Tong University School of Medicine (Shanghai, China) for this study. The rats were housed at 22–25 °C with 60–65% of humidity, under cycles of 12-h light and dark, and with free access to food and water. The health status of rats was examined before the experiment.

Before Sevo treatment, the rat pups were domesticated separately from their mothers. Pups from the same litter were assigned at randomization to two groups. Ten rats were exposed to 100% oxygen at 37 °C in a chamber for 6 h as the control group. The other ten rats were exposed to 2% Sevo at 37 °C in the same chamber for 6 h as the Sevo group. The concentration of Sevo in the room was monitored and maintained by an evaporator. The gas flow into the chamber was 2 l/min. The rats were euthanized after 24-h inhalation or non-inhalation of Sevo for histological study. H&E staining and immunohistochemistry were conducted to analyze the hippocampal tissue.

In addition, for drug intervention experiment, before Sevo treatment, the following adenovirus particles (20 μl, the virus titer was 1.01 × 10^5^ pfu/ml) and ERK/Nrf2 pathway inhibitor ML385 (30 mg/kg, Medchemexpress, Stockholm, Sweden) were injected into the hippocampus of each hemisphere: mimic negative control (NC), miR-140-3p mimic, mimic NC + ML385, and miR-140-3p mimic + ML385 (10 rats for each group).

### Preparation of adenovirus vectors

The adenovirus vectors [short hairpin RNA (sh)-HTR2A, sh-DNMT1, overexpression (oe)-HTR2A, oe-DNMT1, miR-140-3p mimic, and miR-140-3p inhibitor) and control adenovirus vectors (sh-NC, oe-NC, mimic NC, and inhibitor NC) were supplied by Genechem (Shanghai, China). The adenovirus vectors were purified and delivered together with their package vectors into 293FT cells. The supernatant was harvested 48 h later. The adenovirus particles in the supernatant were concentrated at 1:100 by ultracentrifugation and suspended in phosphate buffer saline (PBS) for recovery. The virus titer was determined using enzyme-linked immunosorbent assay kits (Takara, Kyoto, Japan).

### Morris water maze

In order to evaluate the neurodevelopmental outcome of rats, especially the learning and memory function, water maze test was carried out in all groups at the age of 6 weeks (*n* = 10 in each group) as previously described [37]. In short, a circular pool (1.6 m in diameter and 60 cm in height) was used. A submerged platform (10 cm in diameter, 2 cm below the water surface) was placed in a fixed location in the pool with the water temperature set at 23 ± 1 °C. The rats were subjected to a probe test twice a day for 5 consecutive days. The rats were trained to swim and find hidden platforms. The time and swimming distance of rats before reaching the hidden platform were recorded. Following the probe test, the rats were allowed to swim freely for 120 s with the platform removed. The number of times crossing the former platform and the percentage of time spent in the target quadrant were determined. All test data were recorded and analyzed by the MS water maze video analysis system (Chengdu Instrument Ltd., Chengdu, China).

### Step-through test

In order to study the cognitive function during the development of rats, the step-through test was carried out at the age of 3 months, relying on their natural preference to darkness. The devices used in the step-through test contained a behavioral stimulus controller and a video shuttle box (Chengdu Instrument Ltd., Chengdu, China). In short, on the first experimental day, the rats were adapted to dark condition for 2 min, placed in the illuminated compartment, and then re-entered the dark compartment. On the 2nd day, after entering the dark compartment, rats were shocked through the grid floor of the dark compartment. Twenty-four hours later, the retention time of passive avoidance was estimated by comparing the time before rats re-entered the darkroom with any maximum time of 180 s.

### Hematoxylin and eosin (H&E) staining

The hippocampal tissues of rats post different treatments were immersed in 4% paraformaldehyde, embedded in paraffin, and cut into 4-mm sections. The sections were stained with hematoxylin for 5 min, 75% hydrochloric acid alcohol solution for 30 s, eosin for 5 min and 90% ethanol for 35 s. Normal hippocampal neurons had relatively large cell bodies with abundant cytoplasm and one or two large round nuclei. The damaged cells showed contracted cell bodies, dense nuclei, dark cytoplasm and many empty vesicles. The standard semi quantitative scale was used to evaluate the damage of hippocampal neurons. The number 0 indicated no damage to any hippocampus. Grade 1 indicated that hippocampal neurons scattered in CA1 (cornu ammonis) were damaged. Grade 2 indicated that a moderate number of hippocampal neurons were damaged in CA1 subregion (<50% of hippocampal neurons were damaged). Grade 3 indicated that pyramidal cells in CA1 subregion were severely damaged (>50% of the involved cells). Grade 4 indicated extensive cell damage in the hippocampus. Then four random high-power fields of each brain slice were examined. Cells with pyknosis and abnormal morphology were assessed by two pathologists in a double-blind manner.

### Immunohistochemical staining

The hippocampal tissue sections of rats were baked at 60 °C for 20 min, soaked in xylene for 15 min, and dehydrated with graded ethanol. Afterwards, each section was soaked in 3% H_2_O_2_ at ambient temperature for 10 min to block endogenous peroxidase. Following addition of citric acid buffer, the sections were boiled in a microwave oven for 3 min, and subjected to 10-min of antigen repair. The sections were sealed with normal goat serum blocking solution (Sangon, Shanghai, China) at indoor temperature for 20 min, and then incubated overnight with primary antibody to HTR2A (PA5-95288, 1:500, Invitrogen) in the dark. Goat anti-rabbit against immunoglobulin G (IgG) (ab6721, 1:1000, Abcam, Cambridge, UK) was added to the sections on the next day for 30 min of incubation, followed by another 30-min incubation with SABC (Vectorlabs, Burlingame, CA, USA) at 37 °C. Subsequently, the conjugated HTR2A antibody (#ab216959, 1:300, Abcam) was stained with diaminobenzidine (DAB) horseradish peroxidase (HRP) kits (Beyotime, Shanghai, China) at room temperature. The slides were stained with hematoxylin for 30 s, followed by conventional treatments. In addition, all stained images were photographed under observation by a fluorescence microscope (Olympus, Tokyo, Japan).

### TdT-mediated dUTP-biotin nick end-labeling (TUNEL) staining

TUNEL method was adopted to label the apoptotic cells in hippocampal tissues. First, the tissue sections were dewaxed with xylene, hydrated by reducing the concentration of ethanol, and then treated with 20 μg/ml 20% ethanol protease K for 20 min. The sections were blocked with 3% H_2_O_2_ methanol solution for 10 min and incubated overnight at 4 °C in a labeled reaction mixture carrying terminal deoxynucleotidyl transferase and deoxynucleotides. After incubation, all sections were incubated with HRP (POD, 1:500) for 30 min. The sections were treated with DAB solution (30 mg DAB and 200 μl H_2_O_2_/100 ml PBS) in the dark for 15 min and re-dyed with hematoxylin for 1 min. Lastly, the sections were dehydrated with graded ethanol, cleaned with xylene, and fixed with a glass cover before microscopic observation of TUNEL-positive cells.

### Culture and transfection of primary rat hippocampal neurons

The hippocampal tissue of Sprague-Dawley rats was dissected within 24 h and cut into small pieces under sterile conditions. Then they were incubated at 37 °C with 5 mM cysteine, 10 units/ml papain and 0.01% DNase I (Sigma-Aldrich, St Louis, MO, USA). The isolated hippocampal tissue was gently resuspended in Dulbecco’s Modified Eagle Medium (Gibco-BRL, Grand Island, NY, USA) and centrifuged. They were then cultured at 37 °C with 95% air and 5% CO_2_. After 24 h, the medium was renewed with serum-free Neuorbasal medium encompassing B27 (Gibco-BRL), 0.5 mML glutamine (Gibco-BRL) and 2 μg/ml gentamicin (Gibco-BRL). Subsequently, the cells were seeded in a dish pre-coated with 0.1 mg/ml Poly-1-lysine (Sigma-Aldrich) at 8.5 × 10^5^ cells/ml. The purity of cultured hippocampal neurons was determined on the 8th day. Immunofluorescence assay was utilized for the indication of the percentage of neurons and astrocytes of NeuN/glial fibrillary acidic protein (GFAP) in the hippocampus. The purity of NeuN-positive hippocampal neurons was detected by counting the NeuN- and GFAP-positive cells in eight separate areas of the two wells. The percentage of hippocampal neurons and astrocytes was 93% and 6%, respectively.

Adenovirus (1 × 10^8^ TU/ml) was used to infect hippocampal neurons to obtain stably infected cell lines.

### Establishment of Sevo cell model

Hippocampal neurons were cultured in vitro for 8 days and treated with Sevo. In short, hippocampal neurons were exposed to Billups-Rothenburg chamber, which bubbled with Sevo at different concentrations (0.5, 1, 2, 4%) in 95% air and 5% CO_2_ at a gas flow rate of 6 l/min for 7–8 min. Then the reaction chamber was sealed at 37 °C for 6 h, and the concentration of Sevo in the gas discharged from the reaction chamber was confirmed using a Datex™ infrared analyzer (Capnomac, Helsinki, Finland). The control cells treated without Sevo did not need to bubble and were incubated in the standard environment.

### 3-(4, 5-dimethylthiazol-2-yl)-2, 5-diphenyltetrazolium bromide (MTT) assay

The hippocampal neurons treated were seeded in a 96-well plate with density of 1 × 10^4^ cells/well. After incubation at 37 °C for 24 h, the viability of hippocampal neurons was detected by MTT assay. The cells were incubated with MTT (5 mg/ml, 20 μl/well were added into 100 μl cell suspension, Beyotime) for 24 h, and then 200 μl Dimethyl sulfoxide was added to dissolve formazan. The optical density was measured at 530 nm with an automatic microplate reader (Bio-Tek, Winooski, VA, USA). After 30-min exposure of each treated sample to 1% Triton X-100, the results were normalized to the supernatant of the repeated cultures.

### Flow cytometry

After 48 h of transfection, the cells were detached by 0.25% trypsin (in the absence of ethylene diamine tetraacetic acid) and placed in a flow tube for centrifugation. As per the manufacturer’s instructions of Alexa Fluor 488 annexin V/propidium iodide (PI) cell apoptosis kit (Thermo Fisher Scientific Inc., Waltham, Massachusetts, USA), the cells were suspended in 100 μl medium and incubated with 5 μl annexin V and 1 μl PI for 15 min. A BD LSR II flow cytometer (BD Biosciences, Franklin Lakes, NJ, USA) was used finally.

### Methylation-specific polymerase chain reaction (MSP)

Genomic DNA was extracted from tissues and cells to be examined, and then modified with bisulfite. The methylation of the modified DNA was detected by MSP. Partial modified total DNA was amplified by PCR with methylated and unmethylated primers of HTR2A gene (for CPG rich islands). The products were analyzed by agarose gel electrophoresis. Image analysis was implemented by gel electrophoresis imaging and image analysis system.

### Reverse transcription quantitative polymerase chain reaction (RT-qPCR)

Referring to the manufacturer’s instructions, total RNA was isolated from tissues or cells using Trizol reagent (Invitrogen). For mRNA detection, cDNA was obtained by reverse transcription employing reverse transcription kits (RR047A, Takara). For miRNA detection, cDNA was generated with the use of miRNA First Strand cDNA Synthesis (tailing reaction) kits (B532451-0020, Sangon). Expression of targets was analyzed by RT-qPCR using SYBR mixed kits (Vazyme Biotech). β-actin was used as the normalizer of mRNA and U6 as the normalizer of miRNA. The expression was analyzed by 2^−ΔΔCt^. The primer sequences are listed in Supplementary Table 1.

### Western blot analysis

Radio-Immunoprecipitation assay (RIPA) cell lysis buffer containing phenylmethylsulfonyl fluoride was used to extract total protein from cells and hippocampal tissues, and incubated on ice for 30 min. After centrifugation at 24,154.1 × *g* and 4 °C for 10 min, the supernatant was extracted. Bicinchoninic acid kits (23225, Pierce, Rockford, IL, USA) were employed to determine the protein concentration of each sample. The protein was transferred to a polyvinylidene fluoride membrane by Trans-Blot Turbo Transfer System (Bio-Rad, Richmond, Cal., USA). The membrane was blocked with 5% imprinted blocking agent (Bio-Rad) for 1 h and probed overnight with antibodies to DNMT1 [#5032, 1:1000, Cell Signaling Technologies (CST), Beverly, MA, USA], HTR2A (PA5-95288, 1:1000, Invitrogen), ERK (1:500, #9102, CST), phosphorylated ERK (1:500, #4370, CST), Nrf2 (1:1000, sc-722, Santa Cruz Biotechnology, Santa Cruz, CA, USA), Bcl-2-Associated X (Bax, 1:1000, ab32503, Abcam), B-cell lymphoma-2 (Bcl-2, 1:1000, ab196495, Abcam), NOD-like receptor family, pyrin domain containing 3 (NLRP3, 1:1000, ab263899, Abcam), speck-like protein (ASC, 1:500, sc-271054, Santa Cruz Biotechnology), Cleaved caspase-3 (1:1000, #9654, CST), Cleaved caspase-1 (1:1000, PA5-105049, Invitrogen), Cleaved N-terminal Gasdermin-D (GSDMD, 1:1000, ab20197, ABclonal, Wuhan, China) and glyceraldehyde-3-phosphate dehydrogenase (1:1000, #ab9485, Abcam) at 4 °C. Then membrane was incubated with IgG-HRP (1:1000, #7076, CST). Electrogenerated chemiluminescence kits were adopted to detect the signal.

### Dual-luciferase reporter assay

The binding sites between miR‑140-3p and DNMT1 were predicted by ENCORI database. The wild type (WT) sequence of binding sites between DNMT1 3’untranslated region and miR‑140-3p was inserted into pmirG10 luciferase reporter vector (Promega, Madison, WI, USA) to create WT plasmid (DNMT1-WT). Meanwhile, the mutant (MUT) plasmid without miR‑140-3p binding sequence (DNMT1-MUT) was generated in the same way. Hippocampal neurons were co-transfected with the reporter plasmid and miR‑140-3p mimic or mimic NC. After 48 h of co-transfection, dual-luciferase reporter gene detection system (Promega) was applied to measuring the luciferase activity.

### RNA immunoprecipitation (RIP) assay

The binding of DNMT1 to AGO2 protein was detected using RIP kits (17-701, Millipore Corp., Billerica, MA, USA). When the confluency of cells in the 6-well plate reached 80–90%, the medium was discarded. The cells were lysed with equal volume of RIPA lysis buffer (P0013B, Beyotime) on ice bath for 5 min. The cells were centrifuged at 32,876.4 × *g* at 4 °C for 10 min, and then the supernatant was obtained. A part of cell extract was taken as Input, and the other part was incubated with antibody for coprecipitation. For each coprecipitation reaction system, 50 μl magnetic beads were washed and then re-rotated in 100 μl RIP Wash Buffer. According to the experiment grouping, 5 μg antibodies were added including rabbit-anti-rat AGO2 (1:100, ab32381, Abcam) and goat anti-rat IgG (1:100, ab205719, Abcam) and mixed at for 30 min. The magnetic bead antibody complex was resuspended in 900 μl RIP Wash Buffer, add 100 μl cell extract was incubated at 4 °C overnight. The samples were placed on a magnetic stand to collect the magnetic bead protein complex. The samples and Input were detached with proteinase K to extract RNA for subsequent PCR detection of DNMT1.

### RNA pull down

Hippocampal neurons were transfected with biotin labeled Bio-NC, Bio-DNMT1-WT and Bio-DNMT1-Mut RNA (50 nM), respectively. After 48 h of transfection, the cells were incubated with specific lysate (20164, Thermo Fisher Scientific Inc.) for 10 min. Then 50 ml of cell lysate was sub-packed. The residual lysates were incubated with M-280 streptavidin beads pre-coated with RNase-free and yeast tRNA (R8759, Sigma-Aldrich) at 4 °C for 3 h. The binding RNA was purified by Trizol, and the enrichment of miR-140-3p was detected by RT-qPCR (Supplementary Table 1).

### Chromatin immunoprecipitation assay (ChIP)

The enrichment of DNMT1 in the promoter region of HTR2A gene was studied using ChIP kits (Millipore Corp.). Small hippocampal neurons in logarithmic growth phase were fixed with 1% formaldehyde for 10 min to make DNA and protein cross-linked. After crosslinking, chromatin was broken randomly by ultrasonic treatment before centrifugation at 13,000 rpm at 4 °C (Part of the DNA fragment was reserved as input). The supernatant was then sub-packaged into three tubes. IgG of normal rats with NC antibody and DNMT1 (1:20, MA5-16169, Invitrogen) were added respectively for overnight incubation at 4 °C. Protein Agarose/Sepharose was utilized to precipitate endogenous DNA-protein complexes. The nonspecific complexes were washed and cross-linked at 65 °C overnight. The DNA fragment was extracted and purified with phenol/chloroform, and the binding of DNMT1 to HTR2A promoter was detected. ChIP primers were designed by related companies.

### Statistical analysis

SPSS 21.0 (IBM Corp. Armonk, NY, USA) was adopted for statistical analysis. The measurement data were summarized as mean ± standard deviation. Kolmogorov–Smirnov test was used to verify the normality of the data. *p* values of all variables greater than 0.05 indicated that the data were normally distributed. Levene’s test was used to test the homogeneity of variance of the data with *p* values of all variables greater than 0.05 indicating the variance. Data meeting these two criteria were analyzed by parametric test [38, 39]. If the data followed normal distribution and homogeneity of variance, unpaired *t*-test was adopted for two group comparisons. One-way or repeated-measures analysis of variance was used for comparison among groups, followed by Tukey’s post hoc test. *p* < 0.05, *p* < 0.01, and *p* < 0.001 were considered to be statistically significant difference.

## Supplementary information

Original Data File

Supplementary material

## Data Availability

The data that supports the findings of this study are available in the manuscript and Supplementary Materials.
