# Supplementary figures and images for: microRNA-140-3p protects hippocampal neuron against pyroptosis to attenuate sevoflurane inhalation-induced post-operative cognitive dysfunction in rats via activation of HTR2A/ERK/Nrf2 axis by targeting DNMT1
Source: Cell Death Discov. 2022 Jun 16;8:290. doi: 10.1038/s41420-022-01068-4 (PMC9203584; doi:10.1038/s41420-022-01068-4)

Figure 2E


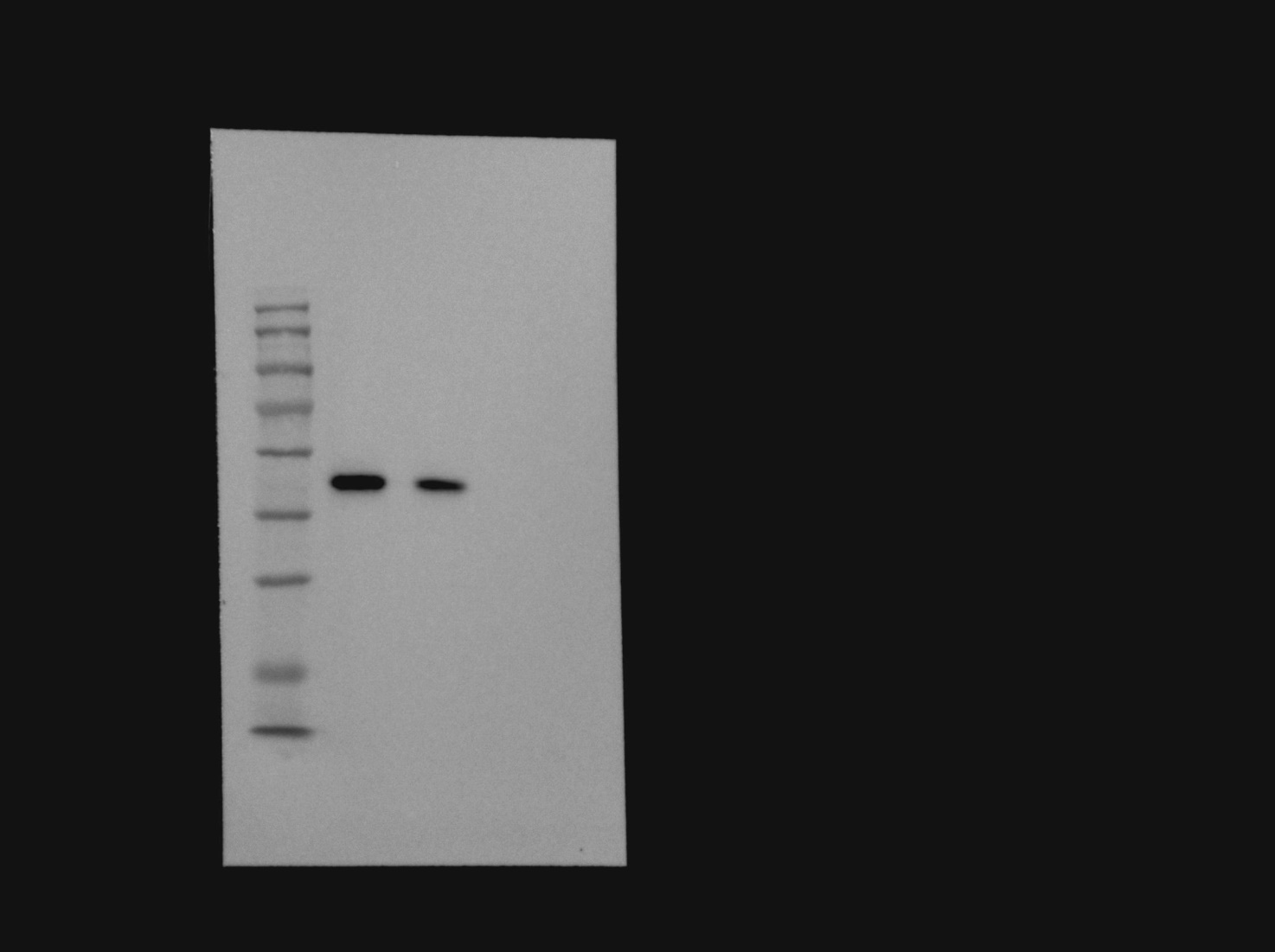


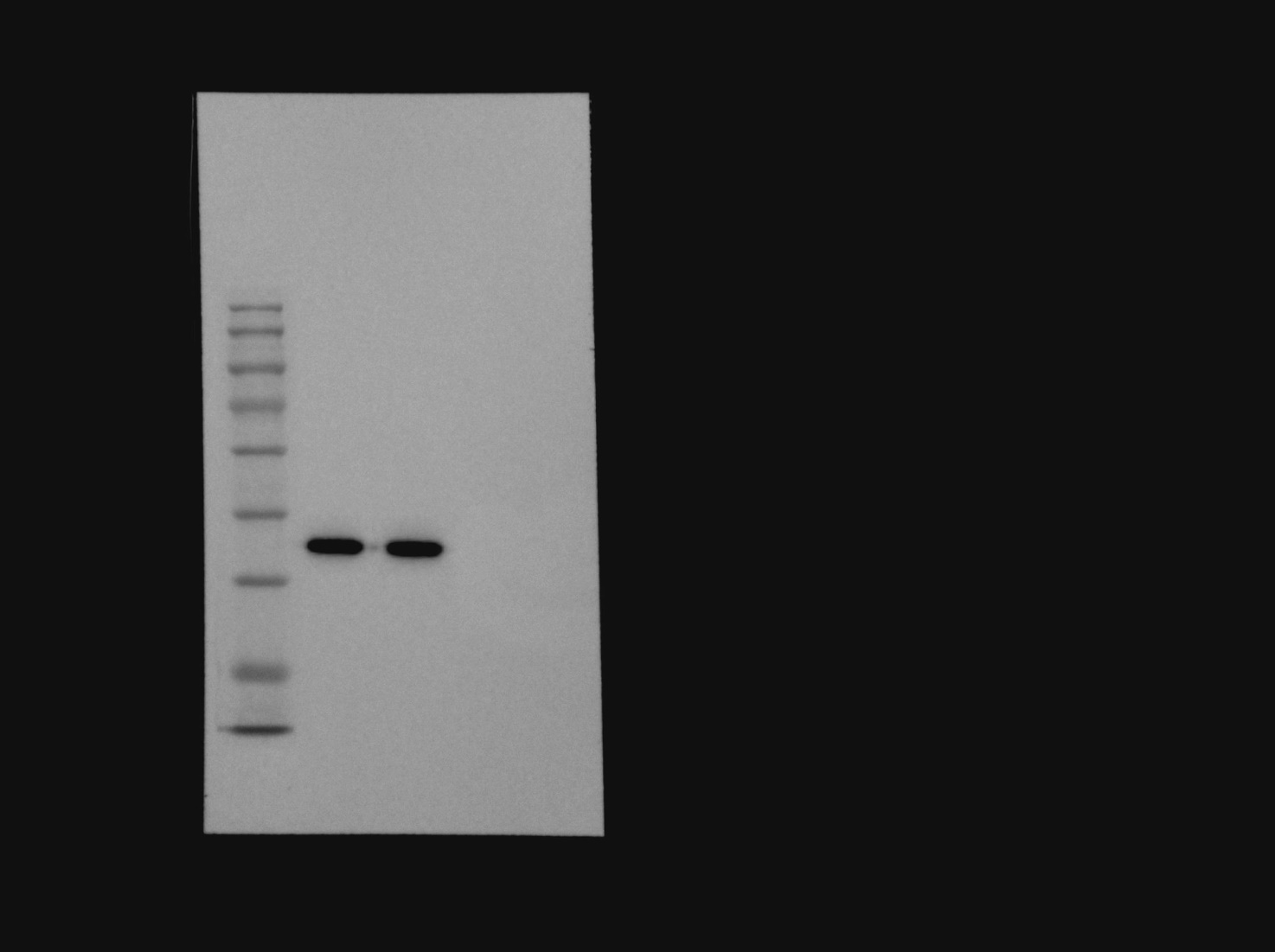


Figure 2H


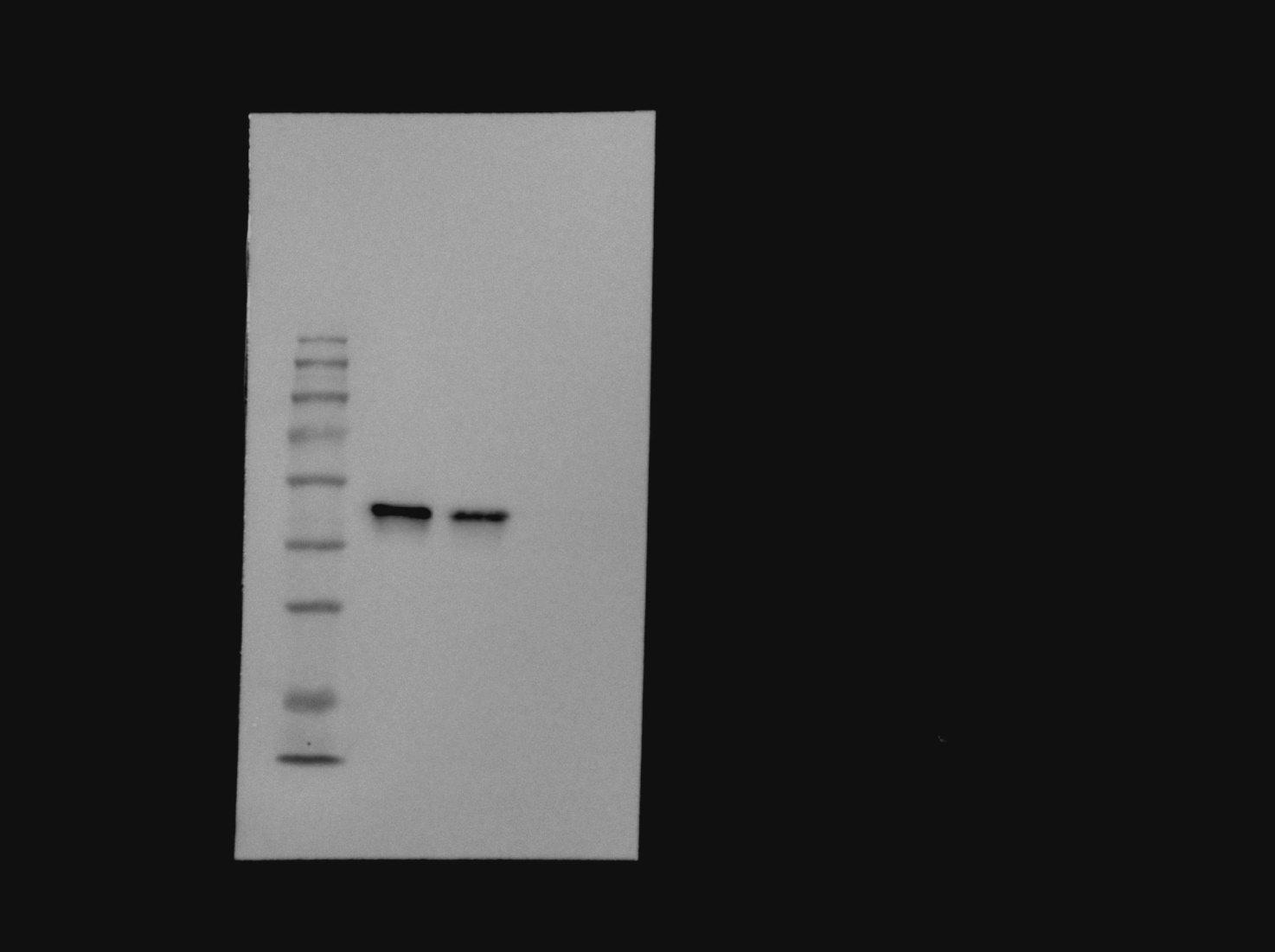


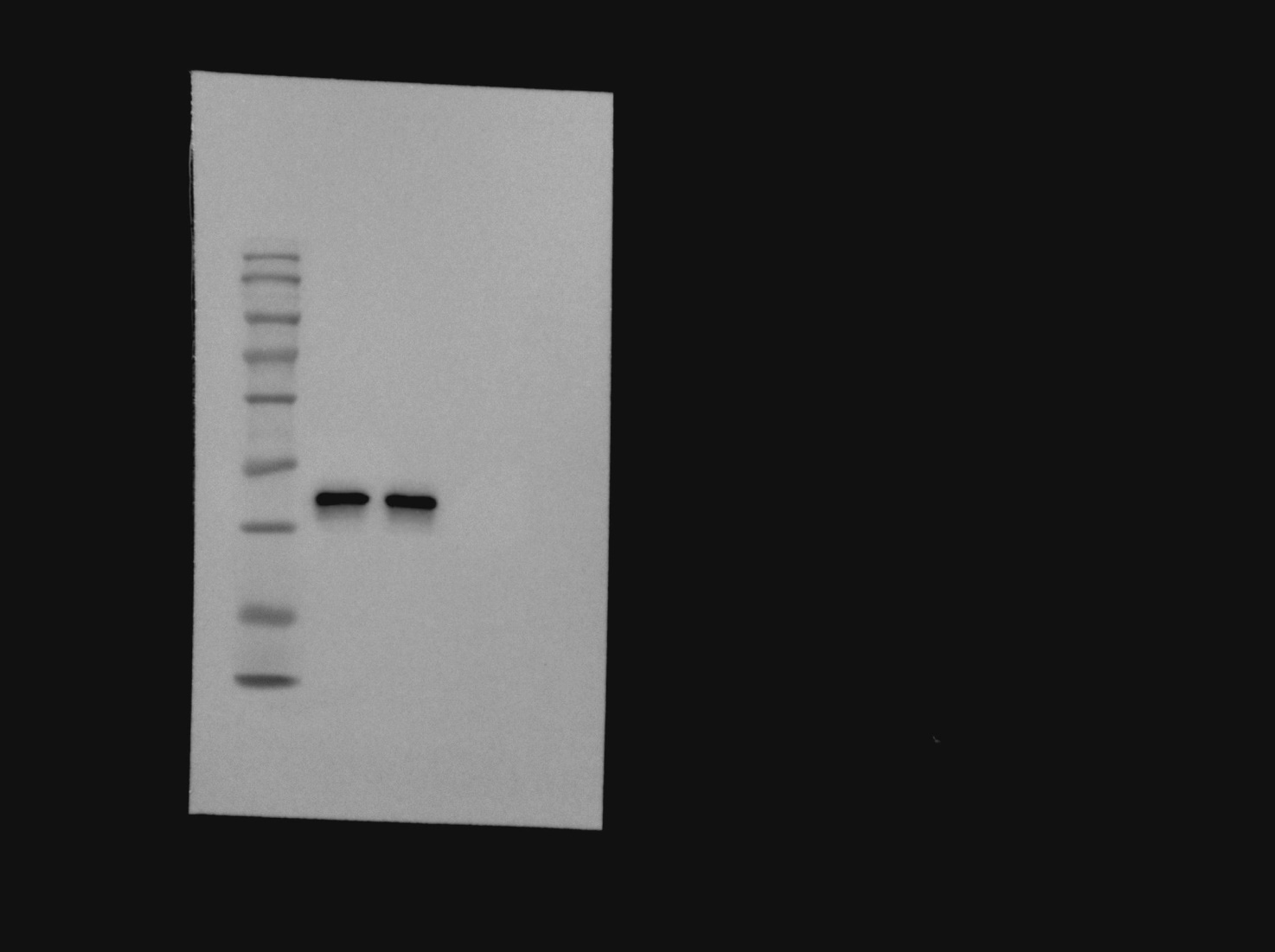


Figure 4L


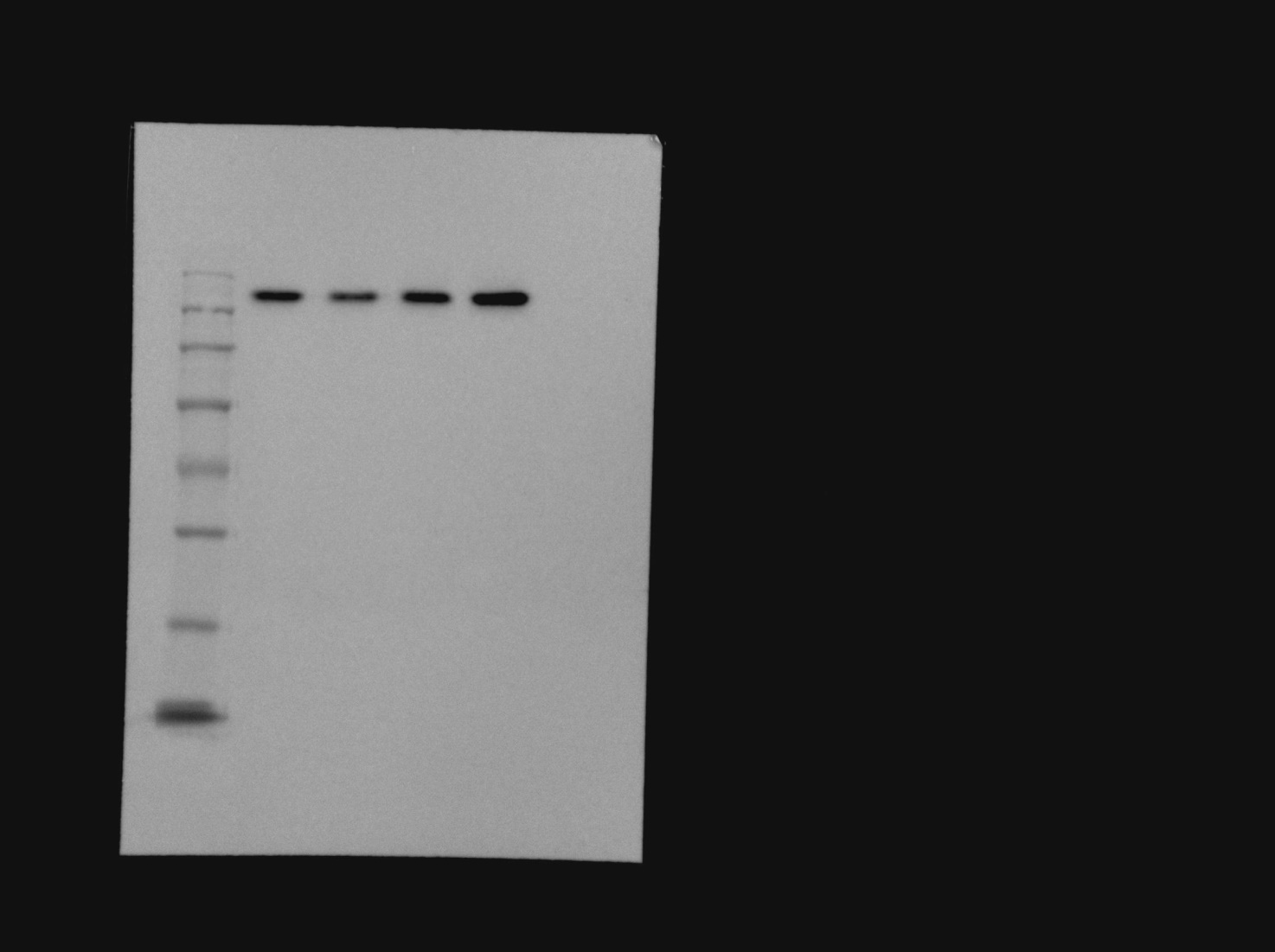


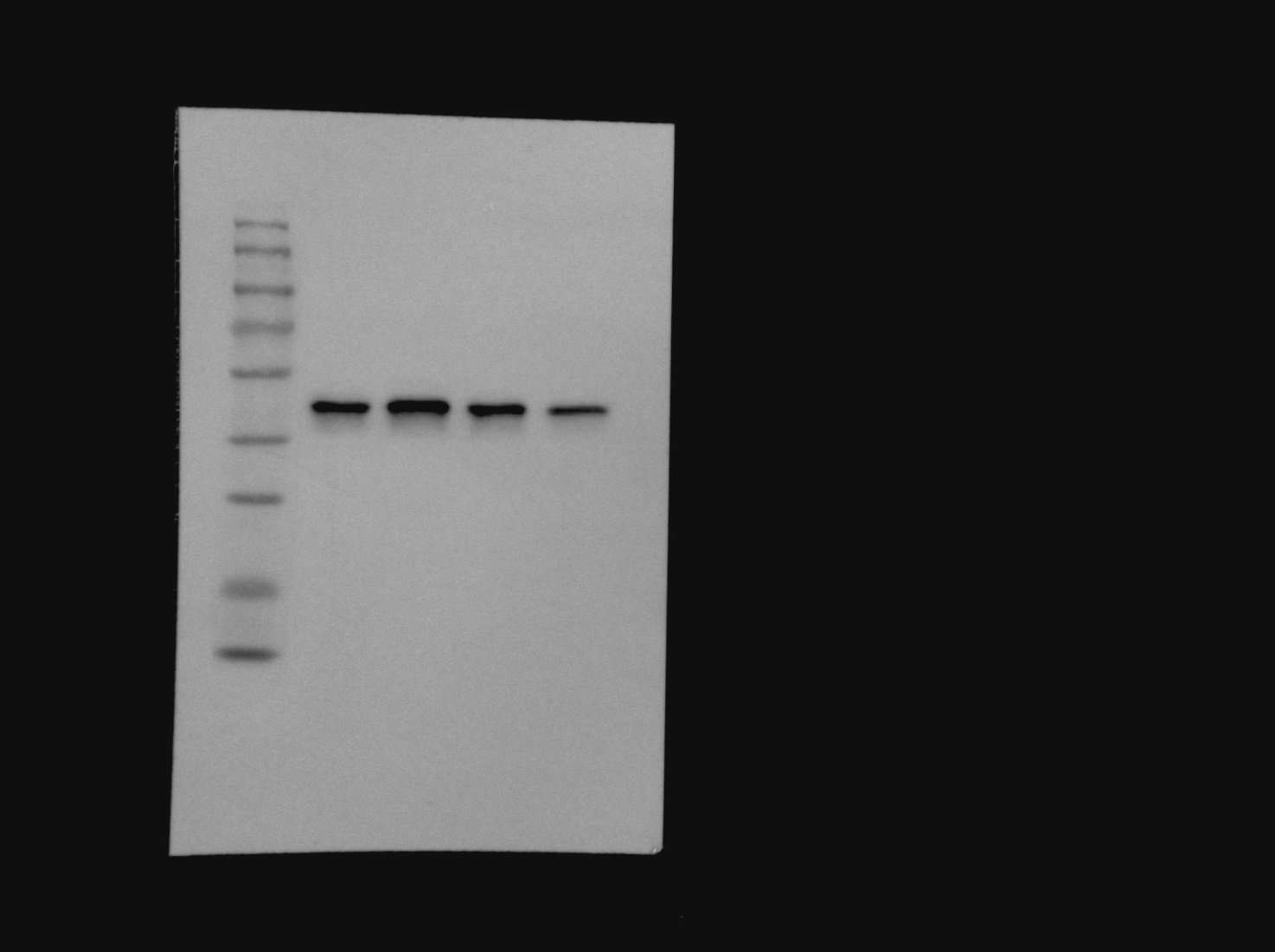


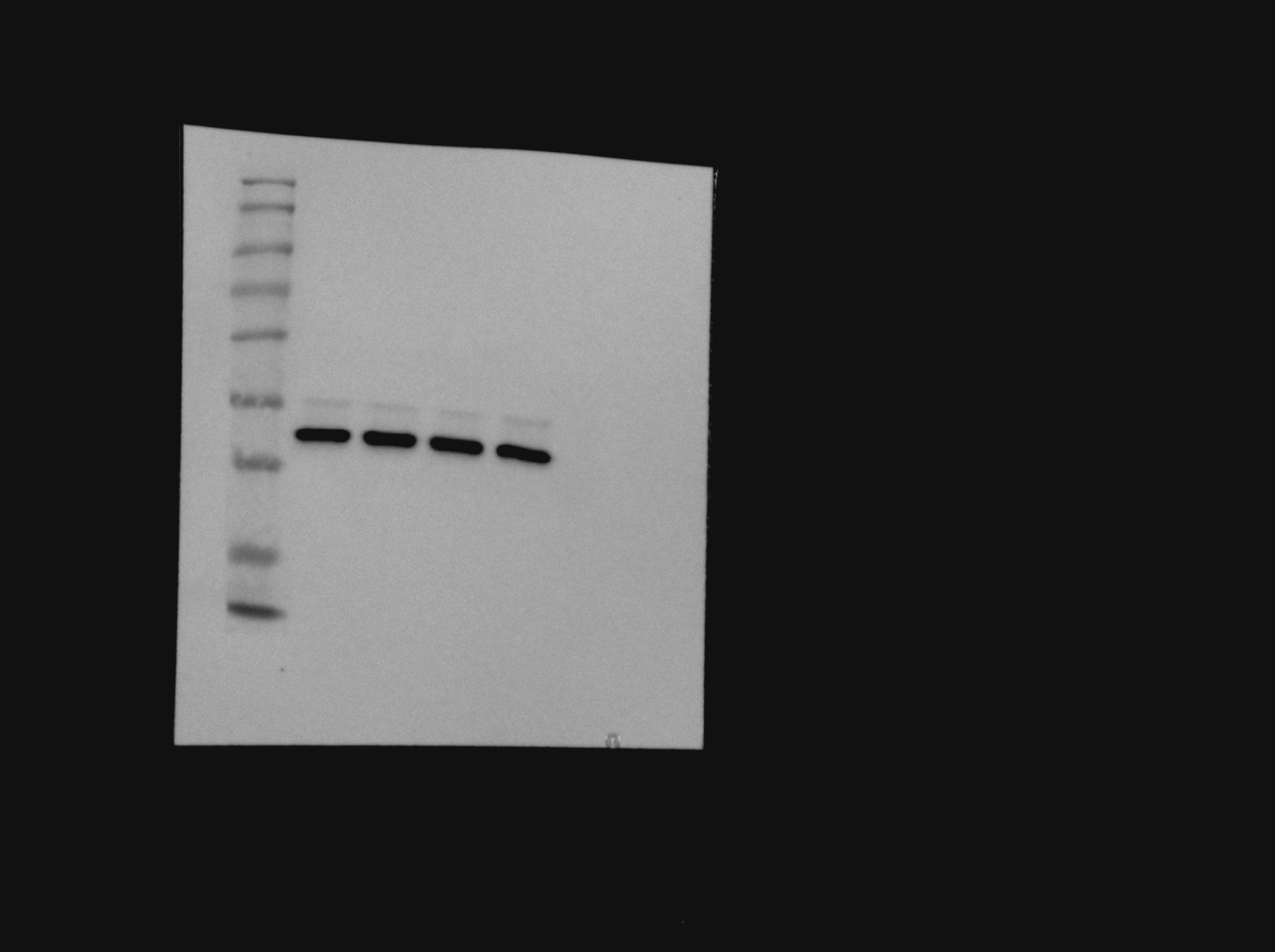


Figure 5J


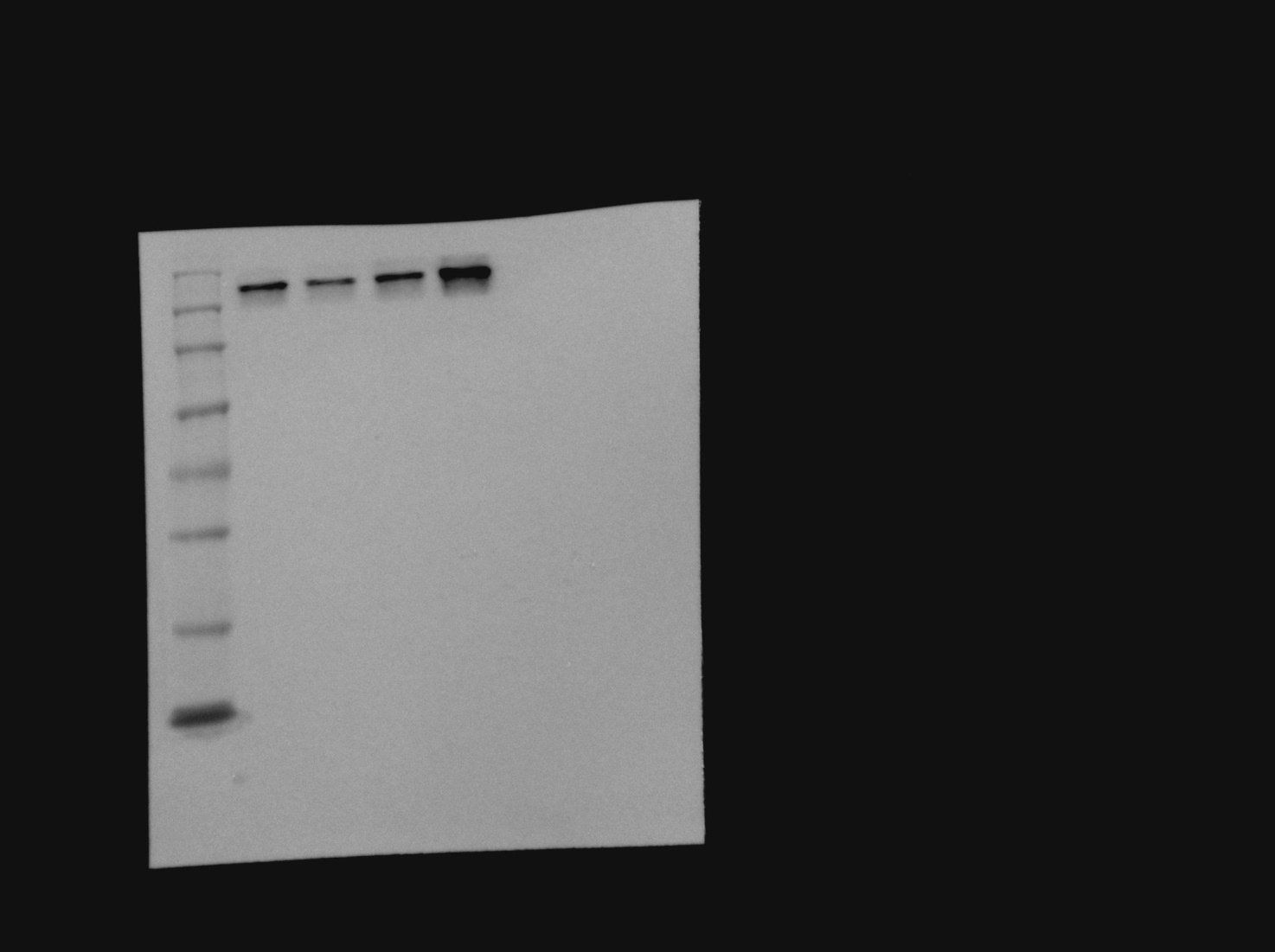


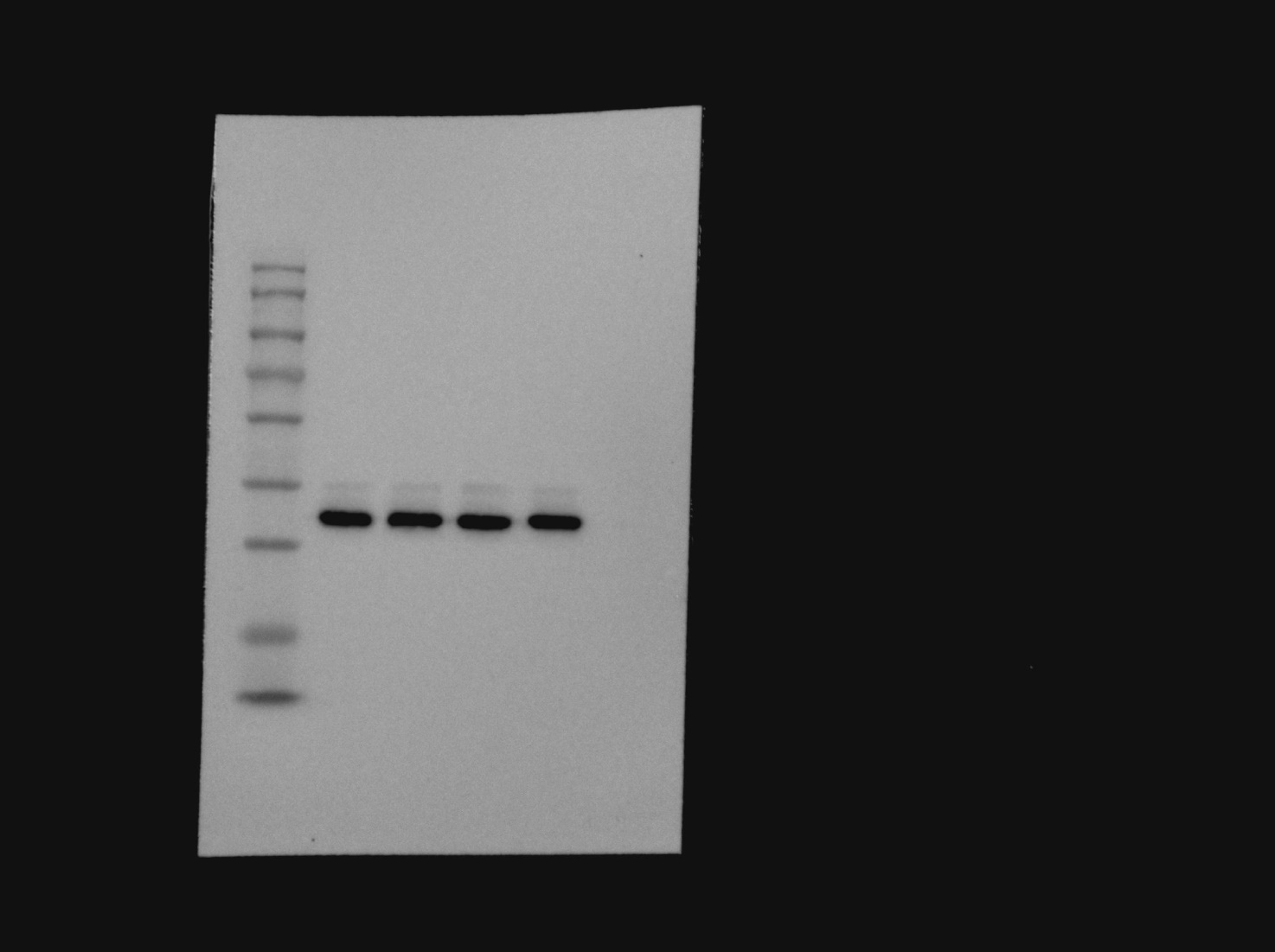

Supplement: Supplementary file 1 — Original Data File [file 41420_2022_1068_MOESM1_ESM.docx]
