# Supplementary material for: microRNA-140-3p protects hippocampal neuron against pyroptosis to attenuate sevoflurane inhalation-induced post-operative cognitive dysfunction in rats via activation of HTR2A/ERK/Nrf2 axis by targeting DNMT1
Source: Cell Death Discov. 2022 Jun 16;8:290. doi: 10.1038/s41420-022-01068-4 (PMC9203584; doi:10.1038/s41420-022-01068-4)
Supplement: Supplementary file 2 — Supplementary material [file 41420_2022_1068_MOESM2_ESM.docx]

**Table S1** Primer sequence of RT-qPCR

| Gene | Sequence |
| --- | --- |
| MSP-HTR2A-M | F: 5’-GATAGGCGAGTTATAGGATAGC-3’ |
|  | R: 5’-AAAAATTCTCACGACAATAACGAC-3’ |
| MSP-HTR2A-U | F: 5’-GATAGGTGAGTTATAGGATAGTGGAT-3’ |
|  | R: 5’-AAAAATTCTCACAACAATAACAACATC-3’ |
| SOD1 | F: 5’-GCAGGGCGTCATTCACTT-3’ |
|  | R: 5’-AGACTCAGACCACATAGGGA-3’ |
| MTHFR | F: 5’-CCTTTCGTGGCCCCAGCTTCAG-3’ |
|  | R: 5’-CACAGAAAGTCCCACGCAGCCA-3’ |
| miR--140-3p | F: 5’-TACCACAGGGTAGAACCACGG-3’ |
|  | R: Reverse universal primer |
| DNMT1 | F: 5’-GGAAAGGAGGAGACTACTAC-3’ |
|  | R: 5’-TCTCACTTGCCACCCACACA-3’ |
| HTR2A | F: 5’-GCTGGGTTTCCTTGTCATGC-3’ |
|  | R: 5’-ACAGATATGGTCCACACGGC-3’ |
| Nrf2 | F: 5’-TGAAGCTCAGCTCGCATTGA-3’ |
|  | R: 5’-TGCTCCAGCTCGACAATGTT-3’ |
| U6 | F: 5’-GTTATGTAGGCACCGCCTTA-3’ |
|  | R: Reverse universal primer |
| β-actin | F: 5’-CCCGCGAGTACAACCTTCTT-3’ |
|  | R: 5’-AACACAGCCTGGATGGCTAC-3’ |
